# Cell-Free Approaches and Therapeutic Biomolecules for Cardiac Regeneration

**DOI:** 10.3390/biom11020161

**Published:** 2021-01-26

**Authors:** Mariann Gyöngyösi

**Affiliations:** Department of Internal Medicine II, Division of Cardiology, Medical University of Vienna, 1090 Vienna, Austria; mariann.gyongyosi@meduniwien.ac.at; Tel.: +43-1-40400-46140

In contrast with some adult human organs, such as liver or skin, the adult human heart shows very limited self-regeneration capacity, attributed to the negligible presence of resident cardiac stem cells or cardiac progenitors. Globally promising therapeutic results in rodent experiments suggested that it could be feasible to replace irreversibly injured myocytes through the delivery of unmatured cells or diverse stem cell types or myofibroblasts. However, in patients with acute myocardial infarction (AMI), cardiac stem cell therapy using intracoronary injection of either autologous or allogeneic cells yielded modest or no improvement in clinical and heart functional outcomes, leading to the suspension of these trial. The increased post-AMI survival rate has led to more patients developing mild to severe ischemic heart failure, generating an urgent need for new therapeutic cardiac regenerative modalities. Due to the complexity of cell-preparation methods, off-the-shelf products have come to the forefront, including new biomolecules, peptides, oligonucleotides, and exosomes. The current Special Issue provides an overview of “cell-free approaches and therapeutic biomolecules for cardiac regeneration”.

In this issue, Verjans et al. discuss the evolution-derived differences in cardiac regeneration among species. They highlight the role of proliferation-capable mononucleated diploid cardiomyocytes in zebrafish and newts (amphibians), which are lost after birth in humans. The authors also discuss the roles played by small and long non-coding RNA families and clusters in cardiomyocyte proliferation and cell cycle regulation, which improve cardiac function. Finally, they summarize the promises and limitations of the modulation of non-coding RNAs for cardiac regeneration [1].

Another article in this issue of Tan et al. focuses on exosomes—small extracellular vesicles that act as inter- and intracellular communicators, thereby conveying cardioprotection and tissue regeneration. The exosomes produced by reparative stem cells, especially mesenchymal stem cells contain several bioactive molecules, such as miRNAs, and may thus serve as a cell-free therapeutic agent. The techniques for exosome isolation and characterization are challenging, and there are not yet standardized protocols. However, due to exosomes’ biological activity in cell-to-cell signaling, they have become a focus of interest in several cell molecular processes, including cardiac regeneration [2].

Nazari-Shafti et al. report the miRNA profiling of extracellular vesicles secreted by mesenchymal stromal cells originated from cord blood or adipose tissue. The authors found that these extracellular vesicles contained several cardioprotective miRNAs, but also large numbers of oncogenic or tumor suppressor miRNAs. Accordingly, they conclude that extracellular vesicles must be subjected to quality assessment prior to human use for cardiac regeneration therapies [3].

In another article of this issue, Zheng et al. summarize the role of the Hippo pathway in cell-free cardiac therapy for ischemic heart disease. Components of the Hippo pathway have influence on cell proliferation, differentiation, and survival; chromatin remodeling of cardiomyocytes; and maintenance of cardiac homeostasis—potentially stimulating the innate immune response of the ischemic injured heart, towards cardiac regeneration. The authors demonstrate that manipulation of the Hippo-YAP pathway can attenuate fibrosis and enable the resolution of post-infarction fibrosis [4].

Mester-Tonczar et al. show that the natural anti-fibrotic compounds bufalin and lycorine moderately reduced ischemia-reperfusion injury in a translational animal model of myocardial infarction. Both compounds exert antifibrotic effects through the deregulation of fibrosis-associated miRNAs. The authors report the first demonstration of the presence and increased overexpression of circular RNAs (circRNAs) in the infarcted pig heart. Moreover, these elevated cardiac tissue circRNA levels were significantly correlated with the left and right ventricular ejection fractions, stroke volume, and infarct size, suggesting that circRNA may play a direct regulatory role in cardiac function [5].

In the article of de Wit et al., the cardiac regeneration processes of different species are compared at the cellular and molecular levels. They describe four different cardiac regeneration processes: cardiomyocyte dedifferentiation, re-differentiation, proliferation, and migration. These processes exhibit species-specific differences and accentuation: e.g., pre-existing cardiomyocytes are dominant in zebrafish; cardiomyocyte proliferation is dominant in newborn mice; and humans predominantly exhibit remaining proliferative capacity and migration, involving several regulatory pathways [6].

Spannbauer et al. contribute a comprehensive overview of the cell-free cardiac regeneration modalities used in translational large animal models. The majority of translational research is performed using pigs due to the similarities between porcine and human cardiovascular systems. The authors provide a detailed discussion of the different substances (proteins, RNA-based therapies, extracellular vesicles, coding and noncoding RNAs, and signaling molecules) that have been administered using diverse delivery modes (intracoronary, percutaneous, or surgical intramyocardial or intravenous administration) [7].

Finally, the review by Garoffolo et al. provides an overview of the cardiomyocyte and extracellular matrix interaction, highlighting the importance of ischemic injury-induced changes in tissues architecture, geometry, and motion (summarized as cellular mechanosensation). These relationships and changes are further discussed in relation to intracellular pathways of gene expressions. Tissue engineered cell-free matrices and scaffolds, with well-characterized chemical and mechanical properties, may represent an option for replacing infarcted scar tissue [8].

In summary, this Special Issue “Cell-Free Approaches and Therapeutic Biomolecules for Cardiac Regeneration” fully covers all fields related to this topic. It includes both comprehensive reviews and original articles that explore different types, delivery modes, and animal models of cell-free cardiac regeneration.
